# Close communication and 2-clubs in corporate networks: Europe 2010

**DOI:** 10.1007/s13278-016-0345-x

**Published:** 2016-06-22

**Authors:** Robert J. Mokken, Eelke M. Heemskerk, Steven Laan

**Affiliations:** 1 Informatics Institute, University of Amsterdam, Amsterdam, The Netherlands; 2 CORPNET, University of Amsterdam, Amsterdam, The Netherlands

**Keywords:** Corporate networks, Interlocking directorates, Close communication, 2-Clubs, Social circle, Hamlet, Coterie, Borough, Pivot

## Abstract

Corporate networks, as induced by interlocking directorates between corporations, provide structures of personal communication between their boards. This paper studies such networks using the framework of a previous paper by Laan et al. (Soc Netw Anal Min, 2016. doi:10.1007/s13278-016-0326-0) where close communication is defined by sub-networks, so that each pair of nodes (boards of a corporation) are either neighbours or have at least one common neighbour. These correspond to sub-graphs of diameter at most 2, designated by us earlier as *2*-*clubs* of three types (coteries, social circles and hamlets), and conform three levels of close communication in social networks. They are all contained within the disjoint *boroughs* of a network, supercommunities which envelope all close communication between nodes of a network. This framework is applied here to an analysis of corporate board interlocks between the top 300 European corporations 2010, using the data from an earlier study by one of us (Heemskerk in Econ Soc 42:74–101, 2013). While the results corroborate the main findings of the earlier studies, our approach also uncovers additional, thus far unrevealed patterns. A single dominant European borough with the Francophone network as its centre and that of Germany only regionally and internally connected. The UK business elite on the other hand is very present and prominent in this European structure of corporate close communication.

## Introduction

Interlocking directorates between corporations exist whenever these share one or more common directors (interlockers) on their boards. For a given set of corporations these interlocks induce a network between its corporations (nodes), where for pairs of nodes (corporations) each common director induces a link between them, which can be represented by a line or edge. In this paper an edge between two nodes (corporations) denotes that they have one or more common directors, so that the network corresponds to a simple graph. Corporate networks are examples of affiliation networks, defined by common membership ties shared by organisations (Wasserman and Faust 1994).

Corporate networks, as induced by interlocking directorates between corporations, provide structures of personal communication between their boards. In such corporate networks interlocks provide channels of personal access and communication by the interlocking directors between the boards of the corporations of which they are a member. A sizeable literature has established that the network of board interlocks facilitates the spread of corporate governance routines and practices from board to board through imitation and learning (among others Davis 1991; Haunschild 1993; Rao and Kumar 1999; Gulati and Westphal 1999; Tuschke et al. 2014). As a communication structure the network of board interlocks provides an opportunity structure for the reproduction of existing beliefs and ideas, as well as for the dissemination of new ones (Heemskerk and Takes 2016).

The large majority of the studies that try to establish how practices and routines spread through networks investigate how two nodes within a dyad influence each other. Typically, they study the likelihood that firm B copies the practices of firm A given that they share a board member. A puzzling finding in the literature is that next to this kind of direct influencing, there is also an indirect influence effect. That is, firm A will be influenced by the practices of firm C, given that they both share a director with firm B (but not with each other). This is particularly prominent in the study of corporate political donations, where indirect board interlock ties between firms are consistently associated with similar donation patterns (Burris 2005; Mizruchi 1989). In other words, firms that share sources of information exhibit unified behaviour.

This implies that the relevant social group within these corporate elite networks must include both direct and indirect neighbours. This has been called *close communication* (Mokken 1980–2011; Laan et al. 2016) and is defined as access and communication between nodes, directly between neighbours (1st step) or through a common neighbour (2nd step). Thus, areas of close communication between boards can be defined as sub-networks where each pair of nodes (corporations) are neighbours or have at least one common neighbour. These can be represented as (sub)-graphs of diameter at most two: each node can reach any other node in one or two steps. For such sub-networks Mokken (Mokken 1979, 1980–2011, 2008) introduced the concept of *2*-*clubs* of a network and its three types (*coterie*, *social circle* and *hamlet*).

In a previous paper Laan et al. (2016) extended this framework with the concept of the disjoint *boroughs* of a network. These boroughs together contain all 2-clubs of a network, forming supercommunities which together envelope all close communication in the network. Recent advances in hardware and corresponding programming techniques provide means and opportunities to find 2-clubs on large networks (e.g. Bourjolly et al. 2000, 2002). Given its theoretical relevance explained above, we apply these concepts to the network of interlocking directorates, using software developed by one of us (Laan 2012, 2014).

One of us gave an elaborate analysis of the network of interlocking directorates of the major European corporations in 2005 and 2010 (Heemskerk 2013; Heemskerk et al. 2013). These studies found that despite the general tendency towards less board interlock activity, the European network of interlocking directorates increased its cohesion. The level of connectedness is rather impressive, as this simple example illustrates. If a member of the board of Deutsche bank would be infected by an extremely contagious flu virus early January, this virus would spread—through shared directors and monthly board meetings—to the boards of over 2100 European firms by late April and to almost 3000 boards by the end of May. While the trend is towards more pan-European interlocking and less intra-national interlocking, the authors point out that by 2010, the European network is still best characterized as a meeting site of several national elites.

We extend Heemskerk’s (2013) analysis of the major European corporations in 2010 from the perspective of close communication. In the following sections we first introduce the conceptual and analytic framework. We then analyse Heemskerk’s network of 286 major European companies, restricting ourselves to its major component of 259 firms. Because this particular dataset is already well studied, it is an excellent case to test the value added of our close community analysis approach.

## Conceptual framework^1^

*Close communication* in a network is defined here as access and communication between nodes, directly between neighbours (1st step) or through a common neighbour (2nd step). *Close communities* in a network demarcate areas where each pair of nodes are neighbours or have a common neighbour. These can be represented as sub-graphs of diameter at most two. According to Mokken (1979, 2008) we represent *close communities* in a network by its 2-clubs, which are *maximal* sub-graphs of diameter at most two: they are not included in or part of another sub-graph of diameter at most two.

Mokken introduced *k*-*clubs* of a network as an alternative to the earlier *k*-*cliques*, which are maximal sets of nodes of a network, such that any two nodes of a *k*-clique are separated by a distance of at most *k* in the network (Luce 1950). However, as noted by Alba (1973), the corresponding sub-graph of a network, induced by a *k*-clique, can have a diameter larger than *k* and even be disconnected. Alba therefore proposed a restriction to just *k*-cliques with diameter *k,* which he called *sociometric clique*. As these were just special and rather coincidental types of *k*-*clubs* among all *k*-clubs of a network, Mokken designated these as *k*-*clans*.

Thus, in this paper close communication in a social network corresponds to *k* = *2*, i.e. 2-clubs, with at most 2-step communication between their nodes. As previously noted in Laan et al. (2016), such personal communication is associated with closely knit groups like cliques, coteries, peer groups, primary groups and face-to-face communities, such as small villages and artist colonies.

Considered as stable dense social sub-networks they can form powerful sources of social capital and support for their members and serve both quick internal diffusion of social innovation and speedy access and exchange of crucial information from outside sources. In the present case of corporate board networks obvious examples are quick interpersonal access and exchange of information and knowledge concerning corporate governance-related actions and practice, e.g. bonus systems, impending fusions or bankruptcies, forthcoming capital floats and governmental actions and lobbying (e.g. Gulati and Westphal 1999).

Moreover, within 2-clubs of a network close reachability of their points involves only the nodes of the 2-club itself, in contrast to 2-cliques, where outside nodes of the network may be required. In that sense, unlike 2-cliques, 2-clubs have a property of local autarchy: the closeness or tightness of their communication structure is independent of the structural relations outside in the larger (super-) network. Hence, any change of that outside structure will not affect the inner structure of a 2-club.

As a consequence close communication within 2-clubs covers very different aspects of social networks than those by other conventional indices, such as the *k*-cores of Seidman (1983)—sub-graphs with minimum degree *k*—or other clustering techniques.

Mokken (1980–2011) showed that there are just three types of 2-clubs, or close communities, conform three levels of close communication.

### Types of 2-clubs

**Fig. 1:**
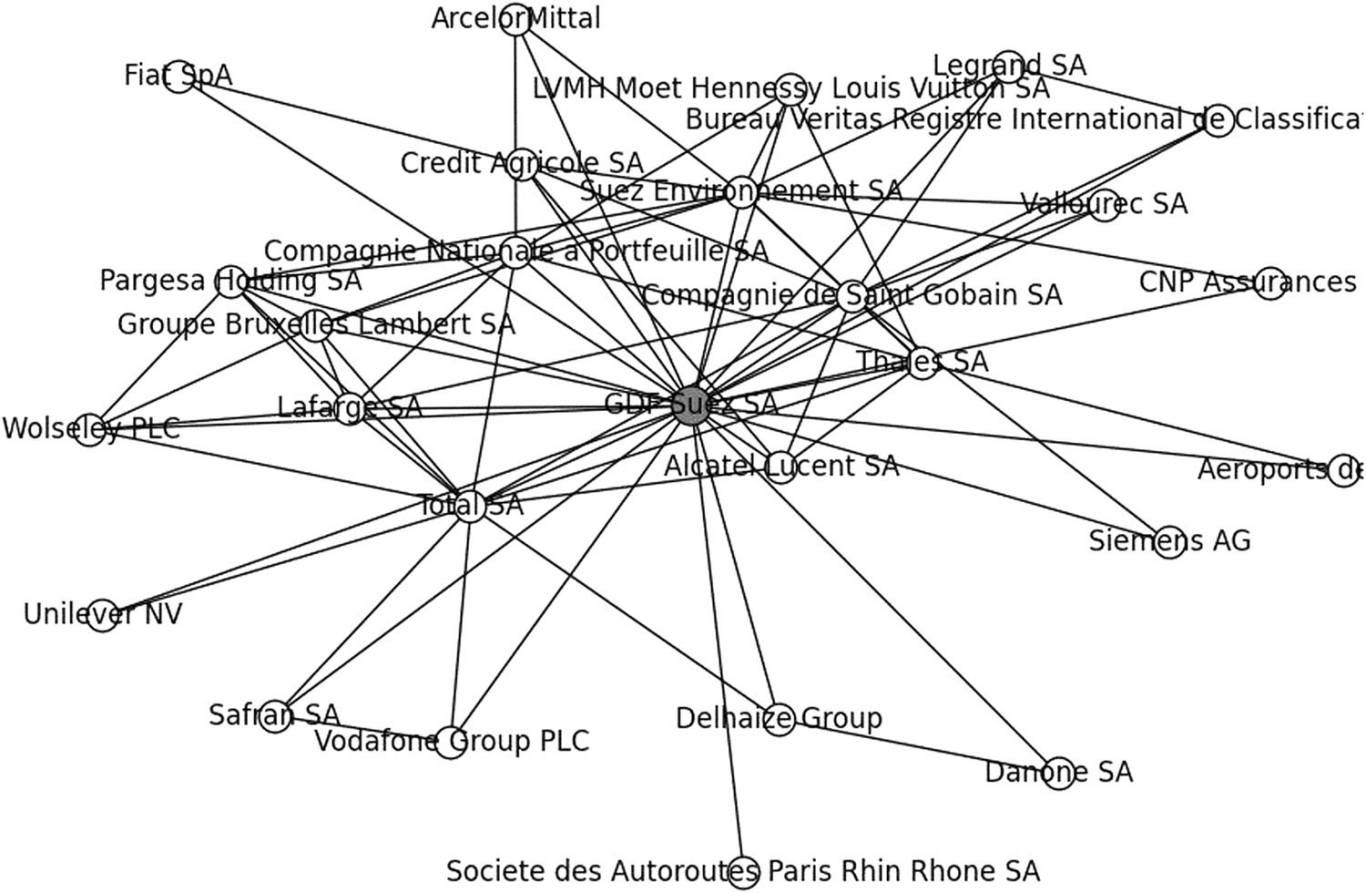
Coterie of GDF Suez SA: 27 firms of which 21 French regional

The *first* type (c*oterie*) corresponds to the ego networks of the nodes of a network (Hanneman and Riddle 2005). For each (non-isolated) node of a network its *ego network* is the sub-graph of that node together with its neighbours and all edges joining them, with the node as single focal point: its centre or ego. Obviously, any ego network has diameter at most 2, but it is only a 2-club, a c*oterie,* if it is not included in any other 2-club of the network. Thus, each coterie has a central or focal node, its ego, which identifies it. Coteries, as all ego networks, are tightly meshed, involving communication along triangles (C3) only, thus confining their level of close communication to strictly local, *within* the ego network around its central point or ego. (An example will be given with Fig. 1). Coteries are less interesting as such. Beyond the fact that they are not included in any other 2-club, close communication is just confined to the ego network of its focal node.

The other two, more proper types of 2-clubs concern a wider setting than strictly local communication within an ego network. They involve close communication *between* ego networks of a network, in a looser, more widely meshed setting.

The second type (social circle) is a 2-club without any central or focal point, but with at least one central pair of neighbours, where each other node of the social circle is neighbour of at least one of the two nodes of that central pair. Consequently, all nodes of a social circle are adjacent to (neighbour of) at least one of the nodes of the central pair. A social circle can have more than one central pairs. We shall see an example in Fig. 3.

Social circles, constituted by triangles (C3) and rectangles (C4), are more loosely meshed. Their level of close communication is confined to an intermediate local level between the ego networks of their central pairs of neighbours. The smallest social circle is 4: a rectangle or cycle of length 4 (C4).

Finally, the *third* type (*hamlet*) is a 2-club without any central node or central pair. Hamlets are constituted by triangles (C3), rectangles (C4) and pentagons (C5) and thus are more widely meshed. Without central nodes or central pairs, they involve close communication between (parts of) ego networks at the widest local level. We shall see later an example in Fig. 6. The minimum size for a hamlet is 5: a cycle of length 5 (C5) or pentagon.

Thus, social circles and hamlets involve close communication *between* ego networks, whereas coteries, as ego networks, just concern communication *within* these ego networks around their central node (ego). Our main focus will therefore be on the social circles and hamlets of a social network.

### Boroughs

The 2-clubs of a connected network are located in its disjoint boroughs (Laan et al. 2016). A *borough* of a connected network is a *maximal* sub-graph with the property that each edge is on a basic cycle [triangle (C3), rectangle (C4) or pentagon (C5)] of the network and therefore also part of one or more 2-clubs of the network. Consequently, each 2-club of a network is located in just one of its boroughs, which roughly can be seen as a collection of overlapping and edge-chained 2-clubs. Boroughs can therefore be seen as collections of all the 2-clubs of a network, forming supercommunities consisting of demarcated dense areas of close communication in that network.

Hence, it should be emphasized that within a borough its 2-clubs can and will overlap heavily in the sense of having many nodes and edges in common.

### Detection and analysis

Although 2-clubs as a theoretical concept were introduced early in the development of social network analysis, for a long time their detection and analysis on larger networks were considered intractable in computational theory (the problem proved to be NP-hard (Bourjolly et al. 2000)) and practice, due to insufficient computational capacity. As a consequence these options were not available in current software packages, such as Ucinet (Borgatti et al. 2002), beyond the limited class of clans, k-cliques, coinciding with *k*-clubs. However, recent computational theory and technology (e.g. parallel processing) have advanced sufficiently for a plethora of algorithmic workarounds and heuristic to appear in the literature (for references see Laan et al. 2016).

As we were not aware of any other reports of actual analysis of 2-clubs for real networks, beyond their detection, we therefore used provisional software to do so, as developed by one of us (Laan 2012, 2014).

The detection of all 2-clubs in a large and dense network will result in a multitude, if not myriad, of mutually heavily overlapping 2-clubs of the three types. To do so for appropriate network datasets we used a two-step approach: first finding the boroughs in the separate components of a network, as the containers of its non-trivial 2-clubs, and then in a second step for each, or selected boroughs to detect and store the 2-clubs contained in them.

Laan (2014) prototype software then stores the 2-clubs in a database, according to type (social circle, hamlet and coterie), which can then be searched and analysed with a provisional front end viewer (alpha version).^2^ This gave us the means to zoom into and analyse selected dense sections of close communication within a borough, such as listing for a node (board of firm or corporation) of the borough the 2-clubs of which it is a member, sorted by type and size, or comparing for a pair of corporations their common or specific 2-club memberships.

This enabled us to derive some additional results for Heemskerk (2013) European corporate dataset for 2010, as illustrated in the following sections.

## European corporate network 2010

Heemskerk (Heemskerk 2013; Heemskerk et al. 2013) gave an extensive comparative analysis of the network of largest stock-listed European companies in 2005 and 2010, as listed in FTSE Eurofirst top 300 index, focussed on the development of this European network between 2005 and 2010 as an economic institutional network, in a period where the political European Union had to cope with the effects of the financial crisis of 2008. In this paper we analyse Heemskerk’s dataset for 2010 solely with the purpose to delve deeper in the close communication areas of this real network and experiment with the associated concepts and methods. For background and details we refer to Heemskerk (2013).

### The European borough 2010

We analysed the data as a simple graph, where the nodes represented firms and a single edge joined two nodes if the firms had at least one interlock, i.e. one or more common directors. Hence, the network is unweighted. The network covered 286 of the major firms and contained a dominant component (maximal biconnected sub-graph) of 259 firms: each pair of nodes in the component is joined by a path in the network.

In this component we found a single dominant borough of 225 firms, in addition to three smaller, trivial boroughs. These coincided with single and disjoint minimal 2-clubs: one of four firms (one British, two Italian and one Spanish) and two of three firms (one of 3 Swiss firms and one of two Spanish and a Portuguese firm).

We confined further analysis to this giant European borough, covering 79 % of all firms in the network and 87 % of those in the dominant component. It is the largest bounded area containing close communities (2-clubs) within the European corporate network of 2010 and its major component. It is packed with (almost) all of its sequentially edge-chained 2-clubs, so that each edge of it is on at least one 2-club. Yet as an area in the network it is rather widely stretched, as its diameter (the longest distance between two of its nodes) is 7.

This European borough contained a total 2128 2-clubs of size 4–7 nodes (firms) and a median size of 15, distributed over the three types as given in Table 1.

**Table 1:** European borough 2010: number and type of 2-clubs, size at least 4

Type of 2-clubs borough	Coteries	Social circle	Hamlet	Total
No. of 2-clubs	138	717	1273	2128
Percentage of total no. 2-clubs of borough (%)	6.5	33.7	59.8	100.0
Size range	4–27	5–25	5–24	4–27
Median size	10	14	16	15
Coverage nodes borough^a^ (%)	99.6	89.4	92.5	100.0

^a^Coverage: % of nodes of borough by nodes in type 2-club

As expected the coteries form a small fraction (6.5 %) of the 2128 2-clubs of the European borough, compared to the social circles (one third: 33.7 %) and the hamlets, which form a majority (59 %) of its 2-clubs. Note that the average size of the three types of 2-clubs, as indicated by their median, increases from 10 for coteries to 14 for social circles and 16 for hamlets, in this sense the largest type of 2-club.

The last row of Table 1 concerning the coverage of the nodes of the borough gives a different picture. With the coverage of a type of 2-club we indicate the percentage of all nodes of the borough that are included in at least one 2-club of that type: thus with a coverage of 99.6 % the nodes in coteries of the borough cover virtually all of its nodes.

In the following three subsections, we shall discuss each of the three types separately.

### Coteries

The ego network of 138 companies, 61 % of the 225 companies in the borough, formed a coterie of the European borough, because they were not part of any larger 2-club of the borough. Together the nodes (companies) in these 138 ego networks cover about all (99.6 %) of the 225 companies of the borough.

The ego networks of the 97 (39 %) other firms were not 2-clubs, because they were included in one or more other 2-clubs.

For instance, the ego network of *Volkswagen AG* was not a coterie but, with degree 12, included in two other 2-clubs of the borough, both hamlets, while *Deutsche Bank AG*, with degree 8, was included in a German social cycle, where it formed one of its two central pairs from *Bayer AG* (the other one with *Deutsche Post AG*).

As to size (the number of their firms/nodes) coteries tended to be smaller than the two other types of 2-clubs: the coteries had median size 10, against a median size of 14 for social circles and 16 for hamlets. For instance, ranked according to size, the 83 2-clubs of sizes 22 or larger counted only four coteries, among 35 social circles and 44 hamlets.

Yet, the largest 2-club in the borough was the French coterie of size 27 with *GDF Suez SA* as its central ego (see Fig. 1). In this ego network *GDF Suez SA* (nowadays *Engie SA*), a French electricity and gas multinational, had 26 neighbours: 16 French, two British, three Belgian, one German, one Italian, one Dutch, one Luxembourg and one Swiss companies. Hence, it was predominantly French regional (together with the francophone Belgian and Luxembourg companies), reaching out to a few non-francophone countries.

For a given 2-club or selected set of nodes (firms) we define its *scope* in the borough as the total number or percentage of all 2-clubs of the borough, each of which contains at least one node of that 2-club or set.

Thus, the ego network of the French company *GDF Suez SA*, both the largest 2-club and coterie of the Borough, had a scope of 1804 of the 2-clubs of the European borough 2010, that is 84.8 % of its 2-clubs shared one or more firms in the coterie (ego network) of *GDF Suez SA*.

Moreover, the six largest coteries, as identified by their central firm, were French:

- *GDF Suez SA* (size 27);
- *Total SA* (size 25);
- *Sanofi Aventis SA* (size 22);
- *Compagnie de Saint Gobain SA* (size 22);
- *LVMH Moet Hennesy Louis Vuitton SA* (size 21);
- *Thales SA* (size 21).

The data for the coteries, as indicated by their central firm, show a correspondence with the findings of Heemskerk et al. (2013, see Table 3). The ranking of the largest coteries by their central firm is similar to the ranking of firms by degree centrality, but different, because, as noted above for *Volkswagen AG* en *Deutsche Bank AG*, they are not counted as a coterie as such, because their ego network is included in another larger 2-club. Thus, ranked by degree centrality *BNP Paribas SA* is listed as fifth most central, in between Saint Gobain and LVMH, while it is absent in the ranking of largest coteries. The firms central to the four largest coteries are also among the top 10 firms ranked by eigenvector and betweenness centrality (Heemskerk et al. 2013). The firms central in the largest coteries are thus able to combine high degree with high betweenness scores, which is in line with our concept of close communication.

We now turn to those close community structures in the European borough 2010, which involve close communication *between* ego networks: its social circles and hamlets.

### Social circles

As introduced above, social circles involve the intermediate level of close communication between ego centres. They are narrowly meshed sub-networks of communication along basic triangles and rectangles. They do not have a single central point, but one or more central pairs of neighbours instead, so that, for each central pair, all other nodes are adjacent to (neighbour of) at least one of its nodes.

There were 717 social circles in the borough (see Table 1), their nodes (firms) covering 89.4 % of its firms. The four largest social circles had size 25 and were French, as they were all spanned by one up to seven central pairs, each involving the pair of *GDF Suez SA* and *Total SA*, the French oil and Gas company, centre of the second largest coterie in the borough, together with central pairs from *Total SA* with other French companies.

**Fig. 2:**
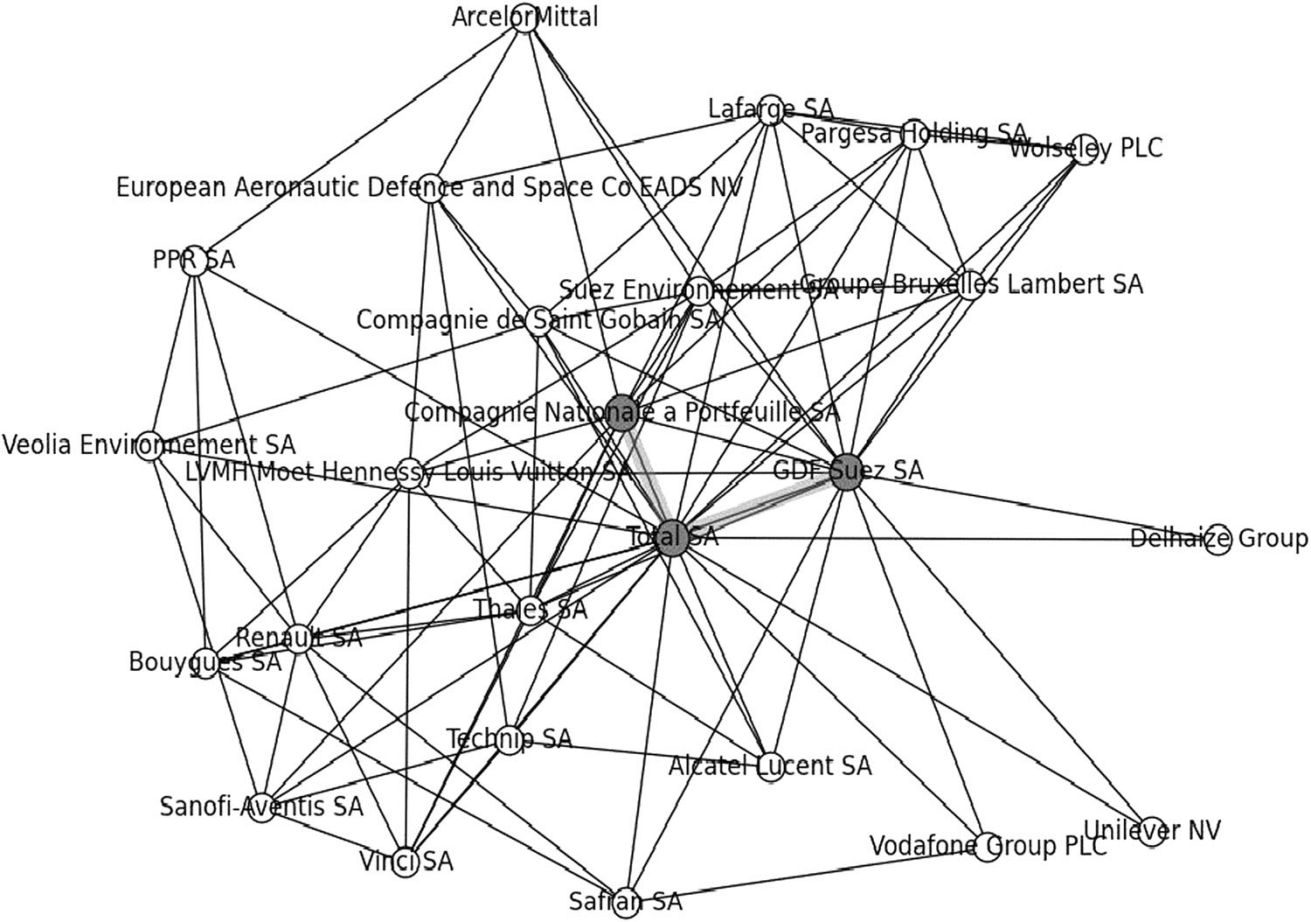
French social circle with two central pairs from total SA

For instance, Fig. 2 illustrates one of these with two central pairs from *Total SA*: one with *GDF Suez SA* and the other with the *Compagnie Nationale à Portefeuille SA*, up to 2011 a francophone Belgian investment holding of the Frère family, which was shown by Laan et al. 2016 to be embedded in the French regional network in close association with *BNP Paribas SA*.

All 35 social circles with sizes larger than 21 were French, in the sense that al their central pairs were formed by the centres of the largest French coteries.

**Fig. 3:**
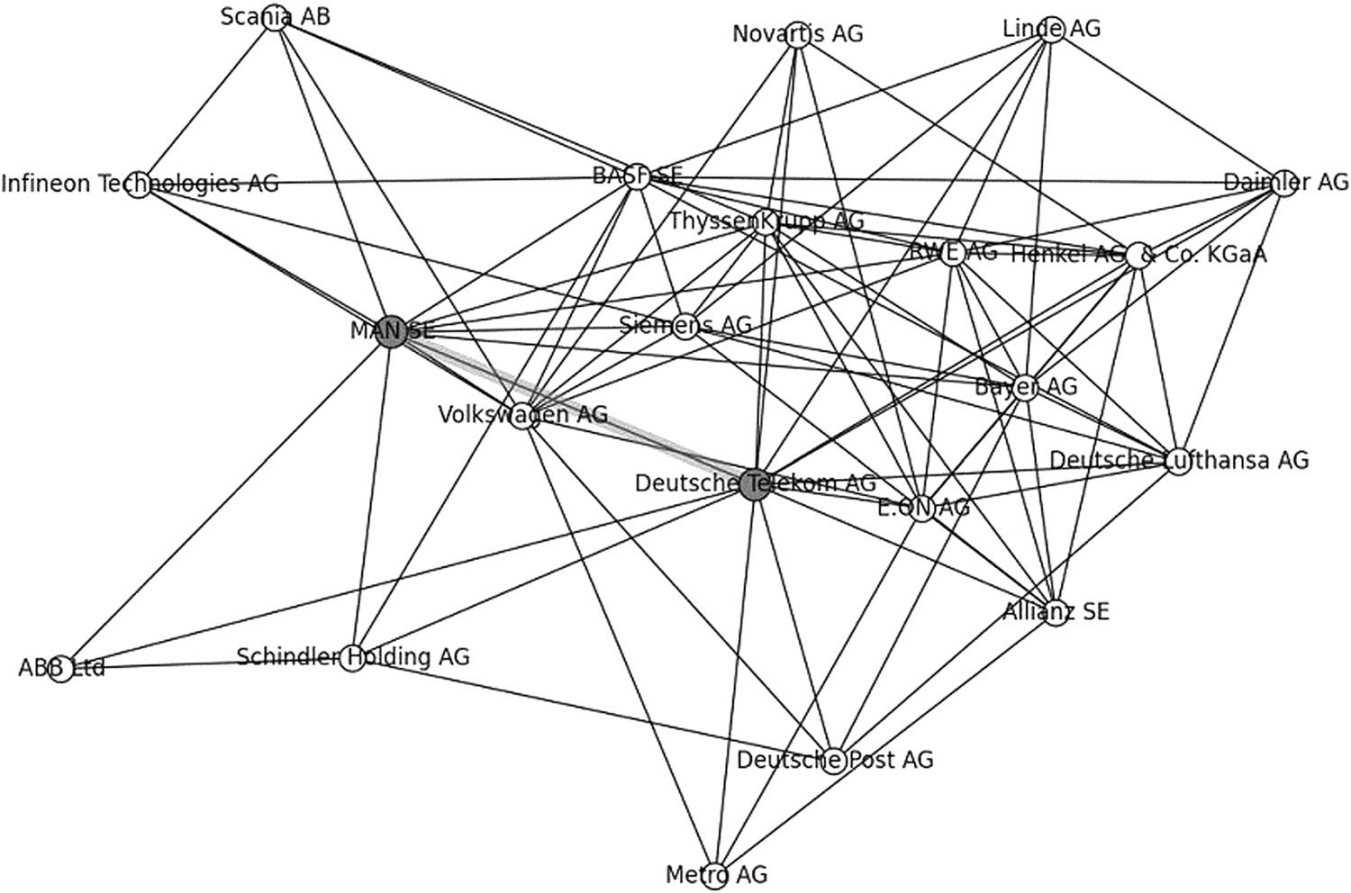
Largest German social circle, 21 nodes: 17 German, 3 Swiss and 1 Swedish

The largest non-French social circle is a German one of size 21 with central pair *Man SE*-*Deutsche Telekom AG* (see Fig. 3). Next to its 17 German firms it has three Swiss firms (*Schindler Holding AG*, *ABB Ltd*, *Novartis AG*) and one Swedish firm (*Scania AB*).

### Hamlets

Hamlets contain close communication at its widest level: widely meshed networks of basic triangles, rectangles or pentagons without a central node or central pair of nodes. The borough contained 1273 hamlets, the nodes of which covered 92.5 % of those of the borough (see Table 1).

The five largest hamlets had size 24. Given the size of their pairwise common overlap (22–23) these represented just two different types, with similar composition. Each type consisted predominantly of the central companies from the French major coteries and a few non-French companies, e.g. two Belgian-French Financials (*Groupe Bruxelles Lambert SA* and *Compagnie Nationale a Portfeuille SA*), two British companies (*AstraZeneca PLC*, *Wolseley PLC* or *Vodafone Group PLC*) and a Swiss financial company (*Pargesa Holding SA*).

Moreover, and similarly, all 44 hamlets of size 22 or larger were predominantly French.

The first largest non-French hamlets are three strongly similar German hamlets of size 21 with large overlaps of 19–20 common firms. The largest predominantly British hamlets are found with sizes 11 or 10.

## National regions and pivotal 2-clubs

As can be seen from the coverage percentages in Table 1, 2-clubs overlap heavily within and between the three types of 2-clubs. Moreover, the analysis in the previous section suggested that the French sphere of close communication appeared rather dominant and dense in the European borough.

In order to focus and sort out other regional spheres of close communication in the borough, we introduce the idea of a common *pivotal* 2-club, or *pivot,* for an adaptively selected set of firms of a certain category, e.g. country, region or industry.

### Procedure of pivot selection

This procedure consisted of an adaptive cumulative selection of firms from a country, according to the size of their coterie or rank in The Global 2000—Forbes.com,^3^ starting with the largest company and downward as long as their set of common 2-clubs is not empty. The final set of common 2-clubs thus is not always unique, containing not one, but possibly a few 2-clubs of different, but usually heavily overlapping 2-clubs. From these a 2-club was designated as a pivotal 2-club or pivot for that set, considered as most representative for that set of firms. Focussing at the widest level of close communication, we chose for a pivot the largest common hamlet, if available. If not present, then the largest hamlet with complete overlap with the largest social circle, else that social circle itself, was chosen.

From this perspective we recapitulate shortly per country or region the results of this procedure in the following subsections, as summarized in Table 2, while referring to pictures of the relevant pivots in “Appendix”.

**Table 2:** Regional pivotal 2-clubs

Region	Type pivot	Size	Scope^a^	
			Total	Percentage of borough (%)
*EU*				
France	Hamlet	22	1618	76.0
Germany	Hamlet	21	627	29.5
UK	Hamlet	11	1724	81.0
Italy	Hamlet	10	791	37.2
Spain	Hamlet	6	72	3.4
Belgium	Hamlet	14	1521	71.5
Netherlands (NL)	Hamlet	12	556	26.1
Sweden	Social circle	15	419	19.7
Denmark	Hamlet	7	277	13.0
Finland	Hamlet	8	449	21.1
*Non-EU*				
Norway	Hamlet	7	33	1.6
Switzerland	Hamlet	8	296	22.5

^a^Scope: number or percentage of 2-clubs of borough containing at least one firm from pivot

### Regional pivots

Heemskerk’s dataset for 2010 covered 16 European countries (Heemskerk 2013). Our European borough for 2010, derived from Heemskerk’s dataset, did not contain any major company from Portugal or Greece (by 2010, no Greek firm shared board interlocks with any of the top 300 firms). The three firms from Luxembourg were all part of French regional networks, as the single Irish firm was part of the British regional networks. Hence, the results summarized in Table 2 refer to 12 European countries: 10 from the European Union and two from non-EU countries.

#### European Union

The first part of Table 2 contains the data for the 10 EU-countries.

##### France

Twelve French companies (the centres of the 12 largest French coteries) and the bank *BNP Paribas SA* had one regionally homogeneous hamlet of size 22 in common, consisting of 21 French companies and one Belgian-French company (*Compagnie Nationale a Portfeuille SA*).

**Fig. 4:**
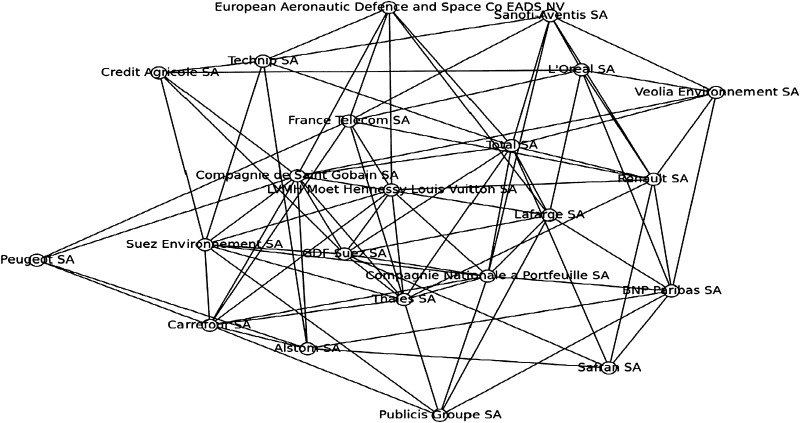
Pivotal French hamlet of 22 French companies

We designated this hamlet as a pivot for the francophone region in the borough (see Fig. 4 in “Appendix”). The *scope* of that French pivot in the European borough, as defined before (see Sect. 3.2), was 1618 2-clubs of the borough, i.e. 76.0 % of the 2128 2-clubs of the borough had one or more firms in that French pivot as well.

##### Germany

**Fig. 5:**
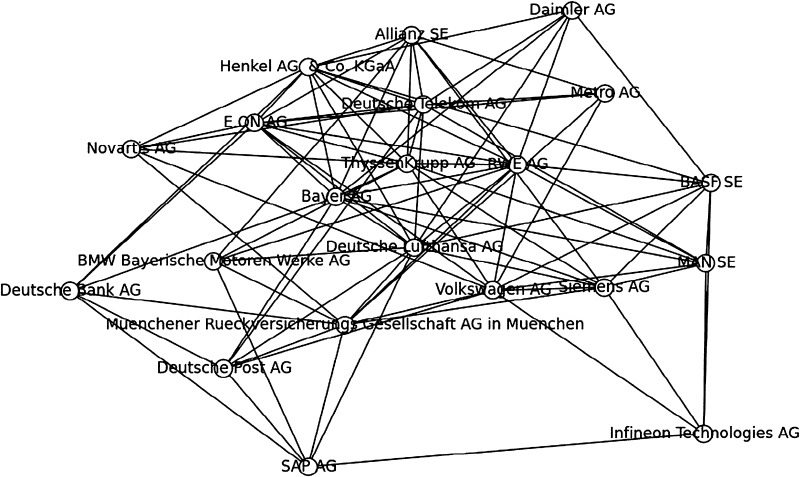
German pivotal hamlet: 20 German companies and 1 Swiss

Twelve German firms, centres of the twelve largest German coteries (sizes 12–18), were all part of just two, almost completely overlapping 2-clubs, both hamlets, each of size 21. One of these was chosen as a representative pivot, for the central part of the German regional network, containing 21 companies, including the largest German bank *Deutsche Bank AG,* and all German companies, except for one Swiss healthcare company *Novartis AG* (see Fig. 5 in “Appendix”).

For the 21 firms of this German pivot Table 2 shows a scope of 627 2-clubs or 29.5 % of the 2-clubs of the borough: 627 2-clubs of the borough had also at least one firm of this German pivot.

Thus, we see that the European borough 2010 contained also a German regional sub-network of close communication, but with a scope of less than half of that of the French regional sub-network.

##### UK

**Fig. 6:**
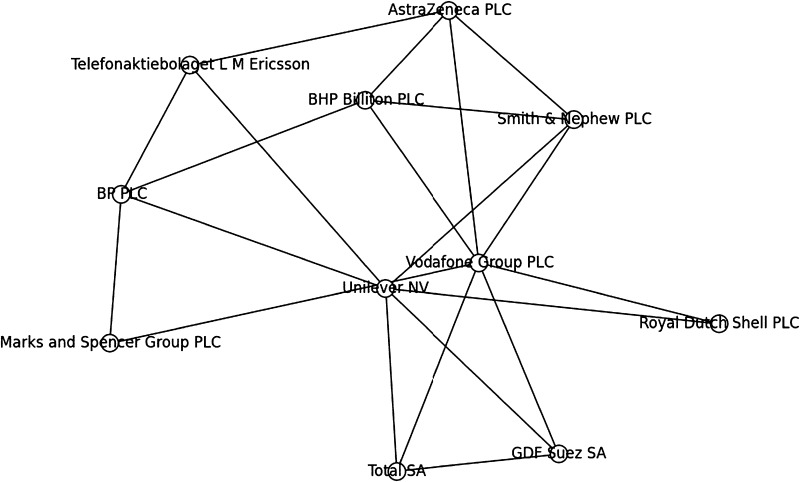
British pivot. Hamlet: 7 British, 2 French, 1 Dutch, 1 Swedish firms

The central companies of the four largest British coteries (sizes 13–20) shared only three 2-clubs, 2 hamlets and a social circle with a mixed Dutch–British central pair *Unilever NV*—*Smith and Nephew PLC*. Taking into account the heavy overlaps we selected the largest hamlet of size 11 (the four largest British and eight other companies) as pivotal for the central part of the British regional network (see Fig. 6 in “Appendix”).

Its 11 companies (7 British, 2 French, 1 Dutch and 1 Swedish) cover 1724 2-clubs of the European corporate borough: each of those 2-clubs has at least one company of that hamlet as a member. Together these 1724 2-clubs form 81.0 % of the 2128 2-clubs of the borough. Note that no British bank was part of that pivot.

Thus, for this “British” pivot, half the size as that for Germany and France, its mixed composition, with four central companies from France (2), the Netherlands (1) and Sweden (1), extends its scope to 81 % of the 2-clubs of the European borough, the largest value in Table 2, and even more than the French pivot (see Table 2).

##### Italy

**Fig. 7:**
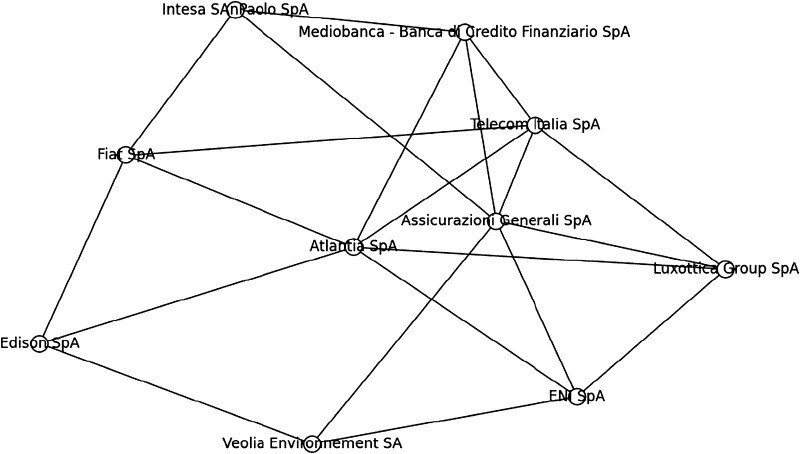
Italian pivot. Hamlet: 9 Italian companies and 1 French

Five of the largest Italian firms, including the bank *Mediobanca*—*Banca di Credito Finanziario SpA*, shared one 2-club, a hamlet of size 10. Apart from these five companies, it contained another four Italian firms and one French firm (*Veolia Environnement SA*). Hence, this hamlet of nine Italian companies and one French can be considered as a pivot for the Italian regional corporate network (see Fig. 7 in “Appendix”).

Together its 10 companies had a scope of 791 2-clubs of the European corporate borough: each of those 2-clubs has at least one company of that hamlet as a member. Together these 791 2-clubs form 37.2 %, of the 2128 2-clubs of the borough (see Table 2).

Thus, its scope, although larger than that of the German pivot, was less than half of that of France.

##### Spain

**Fig. 8:**
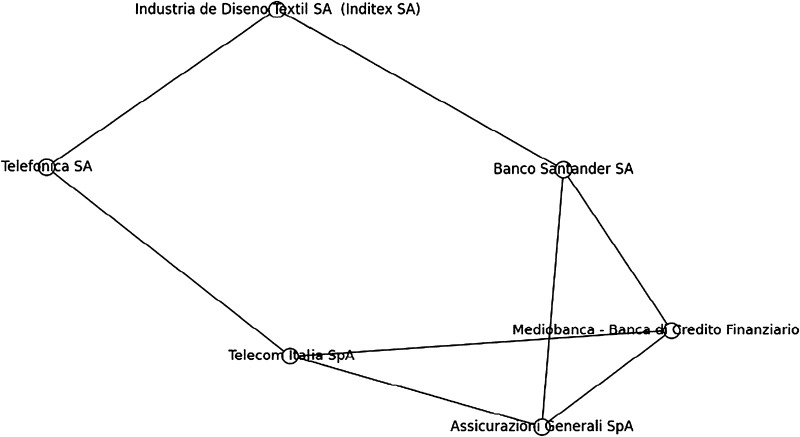
Spanish pivot: hamlet: 3 Spanish and 3 Italian firms

The area of close communication for this large member of the European Union is spectacularly low. A single common, elementary hamlet of size 6 was found, which proved to be mixed binational Southern European, consisting of three Spanish and three Italian companies (see Fig. 8 in “Appendix”).

Its six companies have a scope of only 72 2-clubs, or 3.4 % of the European corporate borough, suggesting a marginal, if not separated position of Spanish regional companies in the close communication areas of that borough (see Table 2).

##### Belgium

**Fig. 9:**
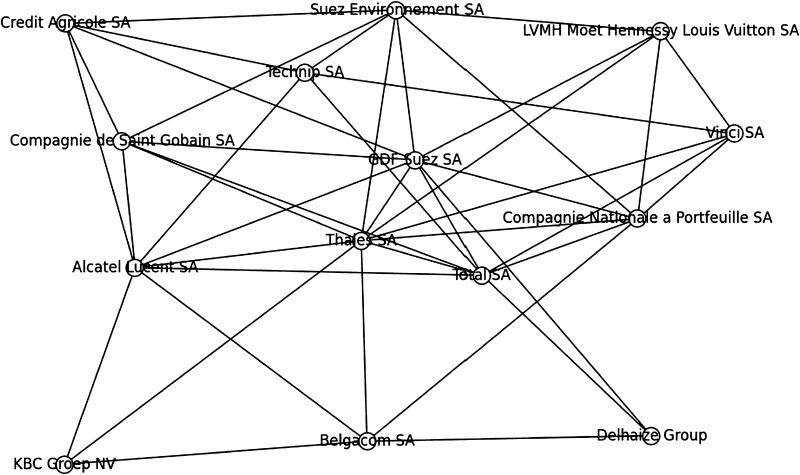
Belgian pivot. Hamlet: 4 Belgian and 10 French firms

Five Belgian companies shared just two 2-clubs: a coterie of size 13 and a social circle of size 9. The social circle (size 9) had central pairs from Belgacom with, respectively, *Alcatel Lucent SA, Thales SA, Compagnie Nationale à Portefeuille SA* and *Delhaize Group*, consisting of five Belgian and four French companies. This social circle was maximally included (but for one firm) in one hamlet of size 14 which we therefore selected as pivotal 2-club for the Belgian region. This pivot consisted of 10 French and four Belgian companies, confirming that this region is mainly part of the larger French regional network (see Fig. 9 in “Appendix”).

Because of that francophone orientation we can see from Table 2 a scope of 71.5 % of the borough for this Belgian pivotal hamlet: its 14 companies access 1360 2-clubs of the European corporate borough: each of those 2-clubs has at least one company of that Belgian hamlet as a member. This scope is more than twice that of the German pivot and indicates that the scope of the Belgian pivot coincides with that of the French pivot.

##### The Netherlands

**Fig. 10:**
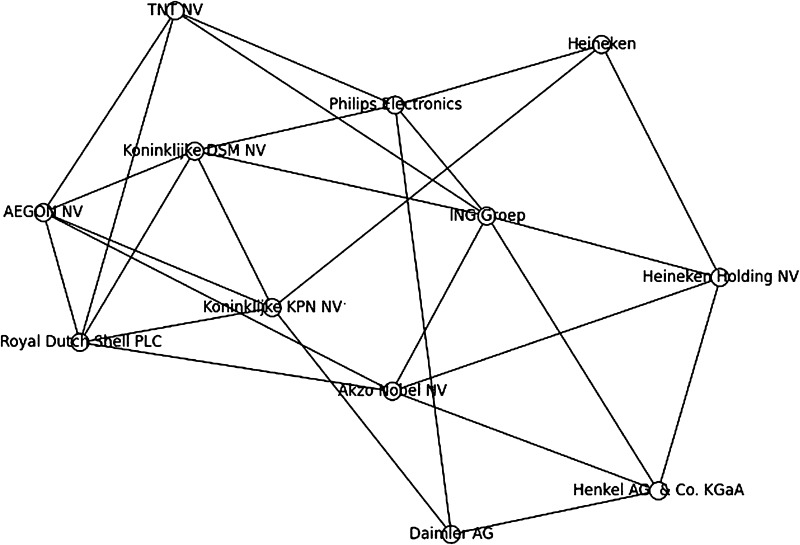
Dutch pivot. 9 Dutch companies and 1 German

Nine Dutch companies are member of just one 2-club: a social circle of size 10, which was maximally overlapped (apart from one firm) by a single hamlet of 12 companies. We chose this hamlet as the Dutch pivot, consisting of 10 Dutch and two German companies (see Fig. 10 in “Appendix”).

From Table 2 we see that this Dutch pivot has a scope of 26.1 %, similar to that of the German regional pivot.

Finally, for the three Scandinavian members of the European Union we found mixed configurations.

##### Sweden

**Fig. 11:**
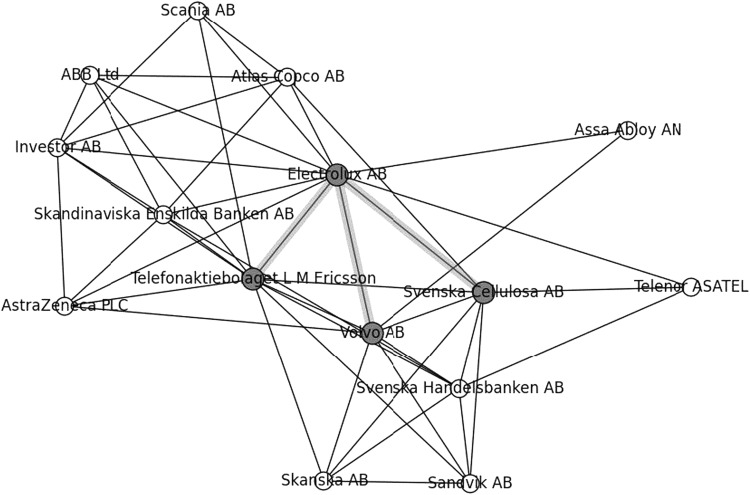
Swedish pivot: social circle with three central pairs. 12 Swedish, I Norwegian, 1 Swiss, 1 British firms

For Sweden we found as pivotal 2-club a single social circle of 15 companies (12 Swedish, one Norwegian, one Swiss and one British). As there were no larger hamlets with maximal overlap we selected this social circle as a pivot of the Swedish regional network. This Swedish regional pivot, less widely meshed than a hamlet, was narrowly organized around three central pairs, all from *Electrolux AB* with *Ericson AB*, *Volvo AB* en *Svenska Cellulosa AB,* respectively (see Fig. 11 in “Appendix”).

Its 15 companies (12 Swedish, one Norwegian, one Swiss and one British) have a scope of 419 2-clubs of the European corporate borough: each of those 2-clubs has at least one company of that social circle as a member. Together these 419 2-clubs form a scope of 19.7 % of the 2128 2-clubs of the borough.

##### Denmark

**Fig. 12:**
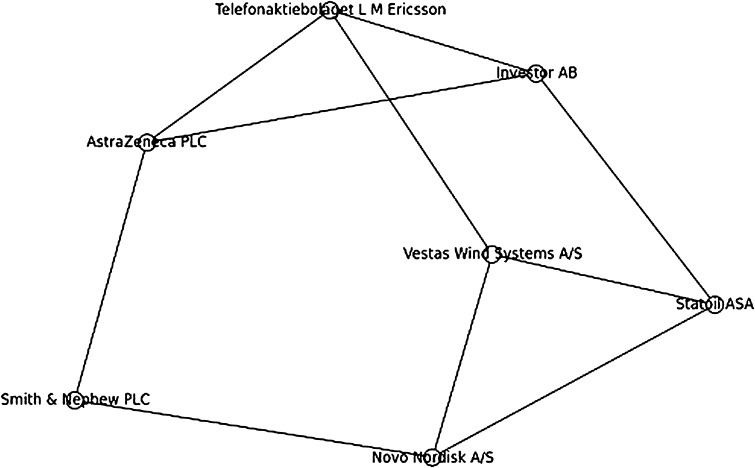
Danish pivot. Hamlet: 2 Danish, 2 Swedish, 1 Norwegian and 2 British firms

For the Danish region we could only find as a pivot an almost minimal hamlet of size 7, consisting of two Danish, two Swedish, one Norwegian and two British companies (see Fig. 12 in “Appendix”). Hence, this hamlet is less a pivot for a Danish region, but more a regional Scandinavian one, supplemented with two British companies. Its seven companies have a modest scope of only 277 2-clubs or 13.0 % of the European corporate borough (See Table 2).

##### Finland

**Fig. 13:**
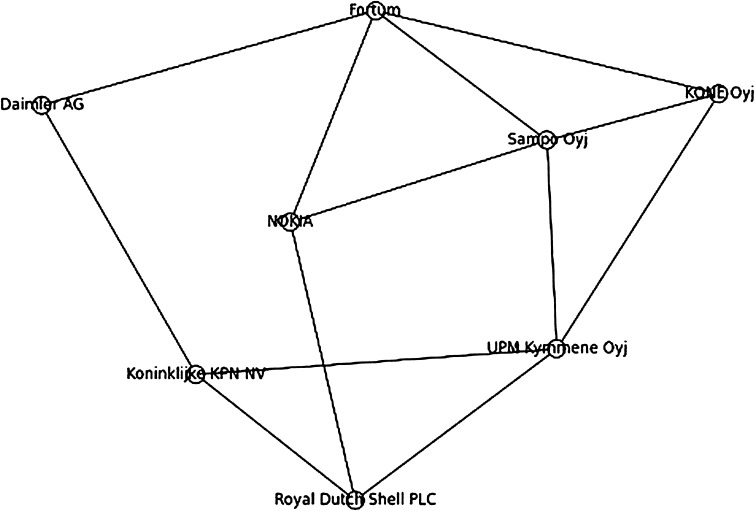
Finnish pivot. Hamlet: 5 Finnish, 2 Dutch, 1 German firms

For all five Finnish companies we found a single common hamlet of size 8, consisting of these five Finnish companies, one German and two Dutch firms (see Fig. 13 in “Appendix”). This mixed hamlet, containing all five Finnish companies, was thus selected as a pivot for the Finnish regional network.

Its eight companies have a scope of 449 or 21.1 % 2-clubs of the European corporate borough: each of those 2-clubs has at least one company of that hamlet as a member. Together these 449 2-clubs form 21.1 % of the 2128 2-clubs of the borough.

#### Non-European Union

At the bottom Table 2 also contains data for two countries outside the European Union: Norway and Switzerland.

##### Norway

**Fig. 14:**
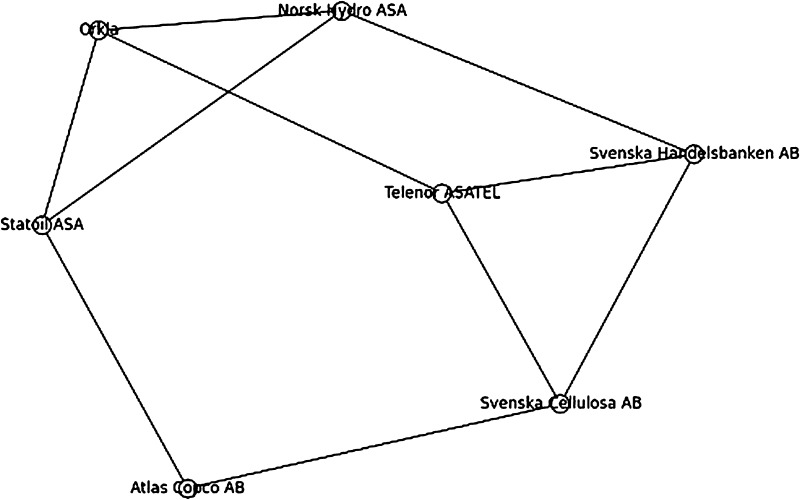
Norwegian pivot. Hamlet: 4 Norwegian and 3 Swedish companies

Four Norwegian companies shared one common 2-club, a hamlet of size 7, with 3 Swedish companies (see Fig. 14 in “Appendix”). As a regional pivot this mixed binational hamlet (4 Norwegian, 3 Swedish) apparently is appended to the Swedish network.

Its seven companies have a scope of only 33 2-clubs or 1.6 % of the European corporate borough, the lowest in Table 2, suggesting an extremely marginal position in the close community structure of the European borough.

##### Switzerland

**Fig. 15:**
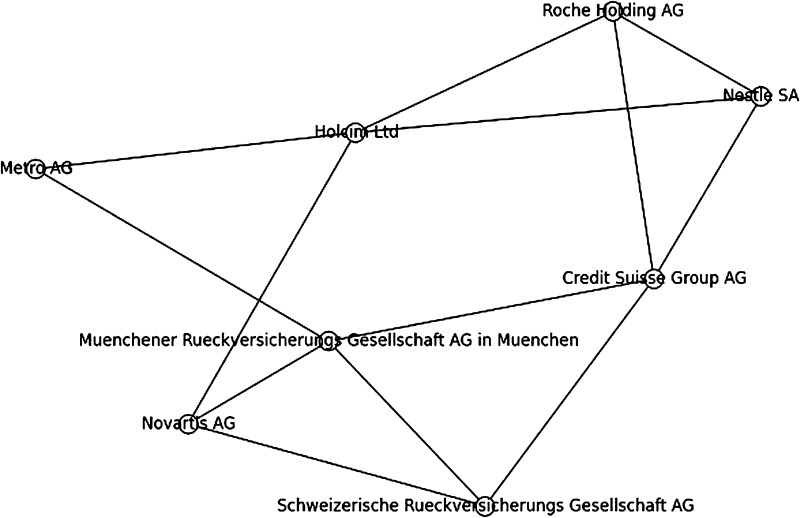
Swiss pivot. Hamlet: 6 Swiss, 2 German companies

Six Swiss and two German companies can be designated as a pivot of the Swiss regional corporate network (see Fig. 15 in “Appendix”). Its eight companies have a scope of 478 2-clubs or 22.5 % of the European corporate borough (see Table 2).

#### Summary

The technique of regional pivotal 2-clubs enabled us to select regional pivots for 12 European countries: 10 of them members of the European Union and 2 non-members. We defined their scope of the borough: the number or percentage of the (2128) 2-clubs of the borough which share at least one firm of a pivot. The scope of a pivot indicates its access to the area of close communication within the corporate network, as determined by the European borough. The high value of 76.0 % for the scope of the large French pivot (size 22) appears to confirm the centrality of the French regional network in the close communication sub-network of that borough. The high scope of 71.5 % for the Belgian pivot (size 14) reflects the inclusion of the francophone Belgian firms in the French regional network.

On the other hand, the moderate scope (29.5 %) of the German regional pivot, with size 21 the second largest pivot, suggests a more peripheral North European position in the European borough.

Similarly, the Italian regional pivot of half that size (10), with scope 37.2 %, suggests a similar marginal South European position in the borough.

Most interesting, if not spectacular (e.g. from the viewpoint of a Brexit), is the position of the British regional pivot. With half the size (11) of the French (22) and German (21) pivots it has the highest scope of all, 81.0 % of the 2-clubs of the European borough, which suggests a much wider range of close communication across that borough.

On the other hand we note the isolated positions suggested by the regional pivots for Spain and Norway with almost minimal size (6 and 7, respectively) and scope: 3.4 and 1.6 %.

These results raise the question of interregional aspects of these regional networks. In the next section we try to get an impression by means of “interlocks” of regional pivots.

## “Interlocking” regional pivots

In Table 2 we noted for some regional pivots, such as the French and German pivots, a homogeneously national composition, for others, e.g. the UK and Denmark, the pivots had a more nationally mixed composition.

This suggests the idea to investigate whether and to what degree these pivots “interlock” pairwise. Two pivots interlock pairwise when they share at least one firm (node) of the network, and the degree of interlock is given by the number of firms in their overlap.

The results are summarized in Table 3. In the second column we see for the pivot of each country the list of other regional pivots, with which they shared at least one firm, and between parentheses the number of firms shared.

**Table 3:** Interlock of regional pivots

Region	Interlocks pivots^a^
*EU*	
France	UK (2), Italy (1), Belgium (4)
Germany	NL (1), Finland (1), Switzerland (3)
UK	France (2), Belgium (2), NL (2), Sweden (2), Denmark (3), Finland (1)
Italy	France (1), Spain (3)
Belgium	France (4), UK (2)
Netherlands (NL)	Germany (1), UK (2), Finland (1)
Sweden	UK (2), Denmark (3)
Denmark	UK (2), Sweden (3), Norway (1)
Finland	Germany (1), UK (1), NL (1)
Spain	Italy (3)
*Non-EU*	
Norway	Denmark (1)
Switzerland	Germany (3)

^a^Number of firms in overlap between parentheses

Again, the UK stands out in Table 3 with the most interlocked pivot of all. Its interlocks cover half the set of regional pivots: France, Belgium, the Netherlands and the three Scandinavian EU member countries (Sweden, Denmark and Finland). Moreover, except for that with Finland (1 firm) most of these interlocks are multiple where the pivot for Denmark with three shared firms appears to be particularly strongly attached to that of the UK.

Notably, there are no interlocks of the British regional pivot with those for Germany and Italy: the German pivot has a solely North European orientation with interlocks with the Dutch, Finnish and Swiss pivots, whereas the pivot for Italy marks a clear Latin European position, interlocking with the French and Spanish pivots only.

With each three common firms, the Swiss and Spanish regional pivots appear to be strongly tied to those of a neighbour: for Switzerland the German pivot and for Spain the pivot of its Mediterranean neighbour Italy.

For non-EU member Norway the pivot is almost isolated from the other 11 regional pivots, sharing just one firm with the mixed Scandinavian pivot of Denmark.

## Discussion

We have re-analysed the well-studied corporate board interlock network of 286 of the largest European companies for the year 2010. We did so from the perspective of close communication areas as defined by its boroughs and 2-clubs, in particular its social circles and more widely meshed hamlets (Mokken 1980–2011, 2008; Laan 2014). We now summarize our findings and relate them to the outcomes of the previous analysis of this dataset.

Apart from three minimally small boroughs/2-clubs (sizes 3–4) the network contained one giant borough of 225 firms, covering 79 % of all firms in the network and 87 % of its dominant component. With a diameter of seven this European borough, containing virtually all close communication between companies in the form of 2128 2-clubs, formed a widely stretched body of close communication in the European corporate network of 2010. As a subset of the dominant component, the diameter of the borough is smaller than that of the dominant component itself, which was nine in 2010 (Heemskerk et al. 2013).

Our approach underscores the findings of Heemskerk (2013) that French firms dominate the network. Analysis of the distribution and composition of the 2-clubs suggested a dense and central dominance of French companies in the borough. For instance, the largest 2-club was a coterie with GDF Suez SA as centre, consisting of 21 francophone companies and six firms from five other countries. Moreover, the 35 largest social circles and the 44 largest hamlets contained predominantly French firms. The results confirm the central position of the French regional level in the European corporate network with a scope covering 76 % of the 2-clubs of the European borough, incorporating the francophone Belgian regional pivot with its scope of 71.5 %. This central position was already foreshadowed by the largest 2-club GDF Suez, which has the largest scope of all: 84.8 %.

Because in a dense network the multitude of 2-clubs is overlapping heavily, we introduced the concept of *regional pivot*, a single 2-club shared by a set of firms from a common region, which enabled us to determine its scope in the borough (percentage of 2-clubs of the borough sharing at least one firm with the pivot) and the interregional, i.e. interpivot structure. We found that the pivots have a distinctive national character, which is in line with the national character of the community structure based on modularity maximization (Heemskerk et al. 2013).

The moderate scope (29.5 %) of the German regional pivot, though with size 21 the second largest pivot, suggested a more peripheral, if not secluded, North European section in the borough. Also notable was the result for Spain: its pivot was just closely tied to Italy, but for the rest with a scope of 3.4 % quite isolated. A similar, peripheral South European position was seen for the Italian pivot. Our findings thus confirm earlier findings that Germany remains relatively light connected, and that Southern Europe is sparsely connected. These similarities with the previous studies increase our confidence about the value of close communities for applied network analysis.

Above and beyond corroborating these previous findings, we find pronounced differences in the 2-club structure across European countries. This suggests that 2-club structure reflects particular varieties of capitalism, such as the Latin (French), the Rhineland (Germany) and the Anglo-Saxon (UK) form. Notable is the profound difference between Germany and France. While both countries are characterized by relatively dense board networks, we show that the French corporations are much better positioned in the 2-club structure of close communication. This finding is in stark contrast with Van der Pijl et al. (2011), who argue that by 2005 German capital has moved to the centre of the network of European corporate interlocks and that German corporations have become nodal points in the communication structures through which the responses to the challenges facing the EU and the West at large are being shaped. Rather, we find that the German close communication structure is inward oriented. Notable in this respect is our finding that banks play a significant role in the German 2-clubs, while this is not the case for the French and British 2-clubs. Bank centrality has been a dominating feature of national networks of interlocking directorates, but banks have never played a key role in transnational or global board interlock networks (Carroll 2010; Heemskerk 2013; Fennema 1982). The enduring importance of banks for organizing close communication structures in Germany suggests that the board interlock network reflects the national business community, rather than a European or transnational business community.

Even more revealing is that while both the German and the French pivots reach out to firms in other European countries, they hardly connect to each other. Hence, the political Franco-German axis that is so crucial in Europe was by 2010 not backed up by close community ties among their respective business elites. This critically challenges the idea of increased cohesion among the European corporate elite as underscored by many recent studies (Carroll 2010; Carroll et al. 2010). Indeed, a recent study of the global network of interlocking directorates among the one million largest global firms suggests that we should consider the network of interlocking directorates increasingly as a “multilevel structure” where, in between the national and the transnational, discernible regional clusters play a fundamental role in the network architecture’ (Heemskerk and Takes 2016, p. 112). Our findings show how close community analysis can be used to better understand such multilevel structures.

Rather spectacular were the results for the British regional pivot: with half the size of the French and German ones, it has the highest scope, 81 %, of all regional pivots, well distributed over the Continental part of the borough. Investigating the interpivot relations in terms of common firms (“pivot interlocks”) we indeed found that by far the most interlocked pivot is that of the UK, covering half the set of regional pivots, but excluding those of Germany and Italy. Contrary to Germany, the UK 2-clubs do not build on financial institutions: it is not the City that drives UK’s centrality. The prominent role of the UK in the close communication structure is a novel finding, not reported by previous studies that analysed this network. Yet, it is consistent with very recent results from a big data network analysis of the board interlocks among the largest 18 million firms worldwide. Looking at the geographical network patterns that connect cities over the globe, they find that the UK community, and more in particular London, is at the centre of the global network of interlocking directorates (Heemskerk et al. 2016). Unlike previous studies, close community analysis identifies this central role of the UK using only a small corporate network.

A recent extensive comparative multicountry and multiperiod study edited by David and Westerhuis (2015), covering the twentieth century and based on *intra*-*national* corporate networks, found for the European countries that from 1980 onward these networks typically fragmented, losing their cohesion and centres. We signalled this process for the Netherlands earlier, when comparing the Dutch corporate network for 1976 and 1996 (Heemskerk et al. 2003). We reanalyzed these data for 1976 and the more recent time point of 2011. That confirmed this result even more clearly: for 1976 we found two boroughs with a dominant borough of size 158, covering 81 % of the main component in the network, and for 2011 seven boroughs, with a main borough of size 94, reduced to 68 % of the main component.

The present study, however, is focused on the *international* European corporate network as such for the year 2010. It suggests that with a dominant borough covering 87 % of its dominant component it shows a much larger cohesion than, e.g. the Dutch corporate network of 2011 and probably most of the other European countries of that period. At the same time we found that it consisted mainly of “interlocking” *national*-*regional* pivots, with a Francophone pivot as a dominant area in the European borough, a German pivot covering mainly its North European part and a British pivot covering even 81 % of it.

Recently Brandes (2016) warned that the rapid growth of the field of network science notably lacks proper reflections on theory and methodology. He suggests network position as an overarching concept that facilitates the development of network analytic procedures and identifies the loci of theory. We see the 2-clubs approach we applied as in line with his call, as we start with conceiving of the position of a node in a network as the entirety of its relevant relationships: directly between neighbours and through a common neighbour.

As such we are confident that the framework of close communication, boroughs and 2-clubs can add new perspectives to the analysis of corporate and other social networks in addition to those offered by the available methods. The application of close community analysis reaches well beyond the empirical example of interlocking directorate networks, ranging from social networks, biological networks, neural networks or infrastructure networks.

## Appendix: Regional pivots

See Figs. 4, 5, 6, 7, 8, 9, 10, 11, 12, 13, 14 and 15.

## Footnotes

1 We assume familiarity with the most basic graph and network notions, e.g. (Wasserman and Faust 1994). For a more complete account see (Laan et al. 2016).

2 A first beta-version of the software is available at https://github.com/Neojume/TwoClubs (Laan 2014).

3 See http://www.forbes.com/…/2010/…/global-2000.
